# Editorial: Role of microglia autophagy in age-related neurodegenerative diseases

**DOI:** 10.3389/fnagi.2023.1175935

**Published:** 2023-03-21

**Authors:** Zihua Wang

**Affiliations:** Fujian Provincial Key Laboratory of Brain Aging and Neurodegenerative Diseases, School of Basic Medical Sciences, Fujian Medical University, Fuzhou, Fujian, China

**Keywords:** autophagy, microglia, neuroinflammation, neurodegenerative diseases, drug delivery

Aggregation of misfolded proteins within cells can result in organelle damage and neuronal dysfunction, which are common pathological hallmarks of neurodegenerative diseases, including Alzheimer's disease (AD), Parkinson's disease (PD), Huntington's disease (HD), and amyotrophic lateral sclerosis (ALS). Autophagy is one of the major mechanisms for the elimination of misfolded proteins and damaged organelles, and has been reported to be involved in the etiopathology of neurodegenerative diseases. Dysregulated autophagy can disrupt cellular homeostasis and lead to various disorders.

Mitochondria are essential for maintaining cellular metabolism and homeostasis, and research has suggested that mitochondrial dysfunction is a contributing factor to the aging process and neurodegenerative diseases. To maintain mitochondrial homeostasis and quality control, organisms employ various strategies, such as mitochondrial biogenesis, fusion, fission, and autophagy, with the latter playing a particularly important role. Wan et al. have reviewed the mechanism and pathway of Sirtuins in controlling mitochondrial quality through mediating mitochondrial autophagy, as well as its role in aging and age-related diseases. Sirtuins act as a protein factor to delay aging and lessen age-related illnesses through a variety of molecular pathways, mainly by encouraging DNA repair, delaying telomere shortening, and mediating the impact of heat restriction on longevity. It is feasible to impede the age-related diseases process by focusing on these pathways.

Microglial autophagy is a mechanism that has been studied in relation to various types of neurodegenerative diseases. Lin M. et al. have reviewed the co-interaction between microglia autophagy and different kinds of neurodegenerative diseases, the potential therapeutic drugs and methods for regulating microglial autophagy to treat or reduce the occurrence and progression of these diseases, including promising nanomedicines. It is suggested that regulating microglial autophagy could be a promising strategy for treating neurodegenerative diseases.

PD is the second most common neurodegenerative disorder, and microglia are the main immune cells in the central nervous system. Recent studies have shown that PD α-Synuclein (α-Syn) can induce the activation of microglia, which participate in the autophagy process through phagocytosis and regulate neuroinflammation, thus providing a neuroprotective effect. The complex autophagy mechanism of microglia in PD has become a topic of interest, and the potential of microglial autophagy as a possible therapeutic target is being explored. Zhu et al. have reviewed the ways of microglial autophagy, discusses the key factors of microglial autophagy in the pathogenesis of Parkinson's disease, and the possibility of microglial autophagy as a potential therapeutic target for PD. Further research into the mechanism of microglial autophagy in PD may provide new strategies for the treatment of the disorder.

Increasing evidence suggests that microglial autophagy is linked to the development of AD and PD, and its impairment is associated with the progression of the diseases. Wang et al. have reviewed the autophagy function of microglia and its malfunction in AD and PD models, and examines the role of microglial autophagy in the pathogenesis of the diseases and its potential as a novel therapeutic target.

AD is a progressive neurodegenerative disorder caused by the accumulation of amyloid β (Aβ) protein, resulting in an imbalance of proteins in the brain. Due to its intricate pathogenesis, it is essential to identify novel therapeutic targets from pathology. Research has revealed that autophagy defects are present in both AD patients and animal models, and that autophagy dysfunction can further lead to oxidative stress, thus increasing the amount of Aβ in AD. It is essential to maintain a normal autophagy level to repair the damage caused by Aβ. Lin H. et al. introduced a transgenic *Caenorhabditis elegans* to study the relationship between autophagy flux and Aβ. The results demonstrated that reducing autophagy accumulation can delay paralysis caused by *Cryptococcus elegans* CL4176 strain and thus exhibit a neuroprotective effect against Aβ-induced toxicity. RNA sequencing and proteomics revealed a variety of autophagy-related signaling pathways, which were affected by Aβ aggregation. In conclusion, managing the normal level of autophagy and lysosome activity, as well as lowering autophagy accumulation, can alleviate Aβ-induced injury and is crucial for the treatment of Alzheimer's disease.

Multiple system atrophy (MSA) is a disorder that can produce Parkinsonian symptoms, as well as cerebellar and autonomic dysfunction, with a range of clinical presentations. The exact cause of MSA is not yet known, however, neuroinflammation may be a factor. Jiang et al. have studied the correlation between inflammatory markers [monocyte to high-density lipoprotein ratio (MHR), neutrophil to lymphocyte ratio (NLR), and red cell distribution width to platelet ratio (RPR)] and MSA. The differences between MSA and Parkinson's disease (PD) were further compared through these inflammatory markers. The MHR, NLR and RPR values of 47 MSA patients, 125 PD patients and 124 healthy controls were tested and the results showed that the MHR, NLR and RPR of MSA group were higher than those of healthy control group. Moreover, the areas under the curve (AUCs) of MSA and PD predicted by MHR was 0.727, indicating that MHR has higher predictive value in the diagnosis and differential diagnosis of MSA than NLR and RPR.

This Research Topic introduces the role and mechanism of microglial autophagy in neurodegenerative diseases, as well as the strategy of targeted cell autophagy regulation. Fruitful achievements have been gained, showing great potential as a therapeutic target for Alzheimer's disease and other neurodegenerative diseases.

## Author contributions

The author confirms being the sole contributor of this work and has approved it for publication.

